# Efficacy and Safety of Intravesical OnabotulinumtoxinA Injection in Patients with Detrusor Hyperactivity and Impaired Contractility

**DOI:** 10.3390/toxins8030082

**Published:** 2016-03-18

**Authors:** Chung-Cheng Wang, Cheng-Ling Lee, Hann-Chorng Kuo

**Affiliations:** 1 Department of Urology, En Chu Kong Hospital, College of Medicine, National Taiwan University, Taipei 23702, Taiwan; ericwcc@ms27.hinet.net; 2 Department of Biomedical Engineering, Chung Yuan Christian University, Chung-Li 32023, Taiwan; 3 Department of Urology, Buddhist Tzu Chi General Hospital, and Tzu Chi University, Hualien 97002, Taiwan; chenglinglee@gmail.com

**Keywords:** underactive detrusor, overactive bladder, onabotulinumtoxinA

## Abstract

We investigated the efficacy and safety of intravesical onabotulinumtoxinA injection in patients with detrusor hyperactivity and impaired contractility (DHIC). Twenty-one patients with urodynamically proven DHIC and 21 age-matched patients with overactive bladder (OAB) with urodynamic detrusor overactivity were treated with intravesical injections of 100 U of onabotulinumtoxinA. The overactive bladder symptom score, urgency severity score, patient perception of bladder condition, global response assessment, voiding diary, and procedure-related adverse events (AE) at baseline, two weeks, one, three, and six months after treatment were assessed. The results showed that the subjective symptom scores improved significantly in both groups, and the scores did not differ between the groups. The decrease in urgency episodes and urgency urinary incontinence were noted in OAB patients but not in DHIC patients. Although the incidence of AEs was comparable between the groups, the therapeutic efficacy lasted for a mean of 4.9 ± 4.8 months in DHIC patients and 7.2 ± 3.3 months in OAB patients (*p* = 0.03). We concluded that the efficacy of intravesical onabotulinumtoxinA injection for DHIC patients was limited and short-term. Nevertheless, AEs did not increase in DHIC. Intravesical onabotulinumtoxinA might not be a good indication in patients with DHIC and high post-voiding residual urine. Physicians should inform patients of the potential benefits and risks of onabotulinumtoxinA injection for treatment of DHIC.

## 1. Introduction

Overactive bladder (OAB) is a common condition in the community and is associated with substantial impairment of mental health and health-related quality of life [1]. Underactive bladder (UAB) is characterized by large post-void residual urine (PVR) volume and weak detrusor contractility [2]. Interestingly, some OAB patients have detrusor hyperactivity and impaired contractility (DHIC), resulting in urgency urinary incontinence and voiding difficulty [2]. The incidence of DHIC increases with age [3], but the underlying etiologies of DHIC remain complex. Chronic ischemia and inflammation of the bladder may contribute to DHIC in the elderly population [4]. Oshiro *et al.* have found that chronic urinary retention results in hypoxia and downregulation of connexin 43, a gap junction protein involved in intercellular communication, in the aged bladder that may reduce the contractility of the detrusor [5]. In addition, bladders in diabetic patients may undergo a transition from a compensated to a decompensated state, which means a transition from OAB to UAB [6]. Though these studies provide possible explanation of the pathophysiology of DHIC, adequate treatment remains difficult.

In the last decade, intravesical injection of onabotulinumtoxinA has emerged as an effective and safe treatment for OAB in patients refractory or intolerable to antimuscarinic agents [7]. Several studies have proven that onabotulinumtoxinA significantly improved OAB symptoms and urodynamic parameters in OAB patients [7,8,9,10,11]. However, increased PVR volume and risk of urinary tract infection (UTI) after onabotulinumtoxinA treatment remain concerns among these patients [8]. Large PVR at baseline and low voiding efficiency (voided volume/bladder capacity, VE) (*i.e.*, VE < 67%) are significant predictors of developing adverse events (AE) after intravesical onabotulinumtoxinA injection for OAB treatment [9]. Therefore, onabotulinumtoxinA is not suggested for OAB patients with a PVR of more than 250 mL unless they are willing to accept the risk of acute urinary retention (AUR) and perform a clean intermittent catheterization.

Whether intravesical onabotulinumtoxinA injection for treatment of DHIC is safe and effective remains unknown. We hypothesized that patients with DHIC might have higher AE and poor therapeutic effectiveness after intravesical onabotulinumtoxinA injection. Thus, we investigated the therapeutic efficacy and safety of intravesical onabotulinumtoxinA (100 mL) in OAB patients who had a baseline PVR > 100 mL or VE < 67%. The results of the study might provide evidence for clinicians to select the appropriate DHIC patients for onabotulinumtoxinA treatment.

## 2. Results

### 2.1. Therapeutic Effectiveness

**Figure 1:**
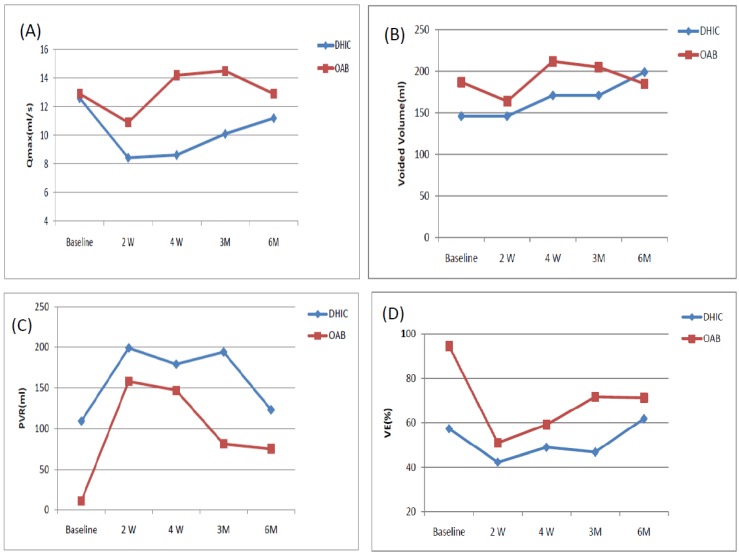
The changes of maximum flow rate (Qmax), voided volume (vol), post-void residual (PVR) volume, and voiding efficiency (VE) at different time-points in patients with detrusor hyperactivity and impaired contractility (DHIC) and overactive bladder (OAB) from baseline to six months. (**A**) Qmax; (**B**) Void volume; (**C**) PVR; (**D**) Voiding efficiency.

The mean ages of the DHIC and OAB patients were comparable (71.2 ± 8.2 *versus* 70.9 ± 14.1 years, *p* = 0.35). Table 1 lists the changes in the variables measured from baseline to six months for the two groups. The subjective symptom scores after onabotulinumtoxinA treatment, including the OAB Symptom Score, Urgency Severity Score, Patient perception of Bladder Condition, and Global Response Assessment all showed significantly improved in both groups. The findings did not differ between the groups. However, the decrease in the number of urgency episodes per three days after treatment occurred only in the OAB patients. Urgency urinary incontinence was significantly improved at three months and six months. Frequency episodes had significantly improved at four weeks and six months in OAB patients, but not in DHIC patients. There was no increase in voided volume in either group. Qmax showed no significant change after treatment in either group. PVR volume increased in DHIC patients at two and four weeks, but not at three and six months after onabotulinumtoxinA injection. However, in OAB patients, the significant increase in PVR volume occurred at two weeks after treatment and lasted for six months. VE showed similar changes to PVR in both groups. The decrease of VE after onabotulinumtoxinA injection was significantly greater in OAB compared to DHIC patients. Figure 1 shows the time-course changes of Qmax, volume, PVR and VE in DHIC and OAB patients.

### 2.2. Adverse Events

Table 2 lists the incidence of AEs in DHIC and OAB patients. The incidence of AEs including AUR, PVR > 200 mL, UTI, gross hematuria and general weakness were all comparable in both groups. After onabotulinumtoxinA treatment, 7 (33.3%) DHIC patients and 16 (76.2%) OAB patients thought that the treatment improved their quality of life. The therapeutic efficacy lasted for a mean of 4.9 ± 4.8 months and 7.2 ± 3.3 months in DHIC and OAB patients (*p* = 0.03), respectively.

## 3. Discussion

To our knowledge, this is the first study reporting the efficacy and safety of intravesical onabotulinumtoxinA injection for treatment of patients with DHIC. We found that patients with DHIC did not have a significantly increased risk of AEs after injection of 100 U of onabotulinumtoxinA compared to OAB patients. However, although the subjective urgency symptom score improved, the number of urgency episodes, frequency or urgency urinary incontinence did not change after onabotulinumtoxinA treatment in DHIC patients. The efficacy of onabotulinumtoxinA therapy declined significantly in DHIC patients compared to OAB patients.

The actual mechanism of DHIC, a paradoxical condition involving both the storage and voiding phases, is not well understood. Clinical observation shows DHIC is commonly associated with chronic bladder outlet obstruction (BOO), diabetes mellitus (DM), neurogenic diseases, and aging [10]. High voiding pressure due to BOO could initially induce OAB. However, chronic high pressure could cause subsequent bladder ischemia and reperfusion injury, which could trigger free radical production injuring the detrusor muscle or neurons [11]. Thus, some BOO patients manifest with DHIC during the transition from OAB to UAB. This phenomenon happens similarly in the DM bladders. Daneshgarin *et al.* have found that DM in the early stage causes compensated bladder function but DM in the late stage results in decompensated bladder function in diabetic animal models [6]. In clinical observations, men with type 2 DM aged <45 years had more OAB symptoms, but adequate voiding function [12]. Voiding dysfunction was commonly found in diabetic patients more than 60 years old, and the duration of diabetes was a risk factor for diabetic bladder dysfunction [13]. Based on these findings, the hypothesis that chronic untreated or treatment-refractory OAB progressed to DHIC and subsequently progressed to UAB with time was proposed [4].

The effective therapeutic choices for OAB include behavioral therapy, antimuscarinic agents, beta-3 agonists, intravesical onabotulinumtoxinA injection, and sacral neuromodulation [14]. However, compared to OAB patients, the treatment of patients with DHIC is often treated empirically, and these treatments often lacked efficacy. Impaired detrusor function limits the use of antimuscarinic agents while options to facilitate voiding function may potentially worsen OAB symptoms or incontinence. Liu *et al.* reported the pharmacotherapeutic outcomes in 43 DHIC patients [15]. Sixteen patients underwent anticholinergic treatment alone, and nine patients had symptomatic improvement. 6 of the 16 patients had symptomatic improvement after alpha-blockers alone. 4 of 5 patients had improvement after a combination of alpha-blockers and anticholinergics. One of the major drawbacks of this study was that the authors did not report how they chose the medication. Schulte-Baukloh H *et al.* reported the outcomes of onabotulinumtoxinA detrusor and external sphincter injection for treating OAB patients [16]. The additional injection of onabotulinumtoxinA into the sphincter reduced the potential risk of patients developing large PVR volumes after injection into the detrusor. This method of injecting both urethral sphincter and detrusor might be helpful for patients with DHIC.

Our study showed that compared to patients with OAB, patients with DHIC had worse therapeutic outcomes, which were proven by the lack of change in the parameters of the 3-day voiding diary. This finding is similar to a previous study of antimuscarinic agents for the treatment of OAB [17]. Hsiao *et al.* [17] showed that failure of the therapeutic efficacy of solifenacin was associated with a low Qmax at baseline and a large PVR volume, which may imply poor bladder emptying in these OAB patients. In a time-course study of diabetic animals, the gene expression of the M2 muscarinic receptors in the urothelium increased significantly by eight-fold at three-week streptozotocin-induced DM bladder and 14-fold at nine-week DM, but only four-fold at 20-week DM [18]. This trend might explain the transition from the compensated to the decompensated stage of bladder urothelial function. The changes in M2 muscarinic receptors in the different stages of DM could have modified urothelial cholinergic autocrine signaling and interrupted barrier function, which could have caused the various therapeutic outcomes found in the clinical studies, regardless of antimuscarinic or onabotulinumtoxinA treatment.

One interesting finding was that, compared with OAB patients, DHIC patients had no significant increase in PVR volumes and AEs after intravesical onabotulinumtoxinA injection. This finding seem different from our previous study showing that the male gender, the baseline PVR volume was ≥100 mL, comorbidities and onabotulinumtoxinA dose > 100 U were risk factors for increasing the incidence of AEs after intravesical onabotulinumtoxinA injection for DO patients [9]. In addition, Liao *et al.* reported an increased risk of large PVR volume and a lower long-term success rate in frail elderly patients with DO after intravesical onabotulinumtoxinA injection [8]. This discrepancy may be explained by our assumption that suburothelial injection of onabotulinumtoxinA might cause less detrusor contractility inhibition compared to detrusor injection and DHIC patients usually voided with abdominal straining. Thus although DHIC patients took more time to empty their bladders, the PVR and Qmax of DHIC could be similar with OAB patients after onabotulinumtoxinA injection. In addition, The VEs at two weeks after treatment (54.1% in OAB *vs.* 42.9% in DHIC) and four weeks post treatment (64.2% in OAB *vs.* 50.2% in DHIC) were comparable in both groups. VE may be an important factor predicting the occurrence of AEs such as AUR, UTI, and PVR volume > 200 mL. Comparable VEs in both groups could have resulted in similar AEs. In addition, we excluded the frail elderly patients from our study. Thus, the effect of the comorbidity on AEs could be decreased in this analysis.

The 2002 ICS standardization report defined detrusor underactivity (DU) as “a contraction of reduced strength and duration, resulting in prolonged bladder emptying and failure to achieve complete bladder emptying within a normal time span” [19]. However, the objective definition of DU based on pressure-flow analyses in clinical studies is not universal. Wang et al defined DU as PdetQmax < 30 cm H_2_O and Qmax < 15 mL/s [20]. Nitti et al defined DU as a BOO index < 20 and a Qmax < 12 mL/s [21]. Jeong et al defined DU as bladder contractility index < 100 [22]. Using a simple method, Resnick *et al.* considered DHIC as involuntary detrusor contractility that emptied less than half of the volume instilled [23]. In this study, we arbitrarily defined DHIC as OAB patients with a baseline PVR > 100 mL or VE < 67%. The ambiguity of the different DHIC definitions requires discussion to achieve a global consensus in the future.

Our study had several limitations. First, the study was limited by its retrospective design and the lack of a placebo control. However, the main purpose of the study was to compare the efficacy and safety of onabotulinumtoxinA treatment in patients with OAB and DHIC. Secondly, the etiologies of patients with DHIC varied and different underlying pathophysiologies of DHIC could have affected the therapeutic outcomes. Finally, all of the OAB and DHIC patients were refractory to behavioral therapy and antimuscarinic agents before onabotulinumtoxinA treatment. The exact therapeutic results of treatment naïve OAB and DHIC patients are still unknown and should be investigated in the future.

## 4. Conclusions

The efficacy of onabotulinumtoxinA intradetrusor injection for treatment of DHIC was limited and short-term. Although the AEs did not significantly increase in DHIC patients, the relatively higher rates of AEs and shorter therapeutic duration still need attention when onabotulinumtoxinA therapy is recommended to patients with DHIC. Intravesical onabotulinumtoxinA might not be indicated in patients with DHIC and high PVR. Physicians should inform patients of the potential benefits and risks of onabotulinumtoxinA therapy for treatment of DHIC to reduce patients’ over-expectations before treatment.

## 5. Materials and Methods

The institutional review board and ethics committee of the Tzu-Chi General hospital approved the study (IRB-094-08), approved 8 May 2014. All participants were informed about the possible AE after onabotulinumtoxinA injection and written informed consent was obtained from all patients before treatment. The clinical trial registration number was NCT-02135341.

A total of 21 patients with videourodynamically proven DHIC and 21 age-matched control OAB patients with urodynamic detrusor overactivity (DO) were retrospectively selected from patients who had participated in previous clinical trials at the authors’ hospital from 2004 to 2009 [9,24,25]. Because this was a retrospective study, we could not reroll patients based on power calculation. Patients of either gender, aged ≥ 20 years of age with videourodynamic DO and, at least, one episode of urgency (urgency severity scale (USS) score ≥ 2) or urgency urinary incontinence per day, as recorded in a 3-day voiding diary, were enrolled. DHIC was defined as the presence of involuntary contraction during the filling phase and underactive detrusor function during voiding phase. Patients with underactive detrusor should fulfill these criteria including PVR > 100 mL, PdetQmax < 30H_2_O, Qmax <15 cm/s and relaxed sphincter EMG without outlet obstruction radiologically during videourodynamic study. We investigated patients with DHIC having a PVR of > 100 mL but < 250 mL or a VE of less than 67% at baseline. Patients with DO had a PVR < 50 mL and a VE > 67% at baseline.

All patients had tried behavioral modification and treatment with antimuscarinic agents for more than 3 months before enrollment. Antimuscarinic drugs were discontinued on the day of screening to wash out the residual effect and obtain a voiding diary that reflected the true bladder condition. Major exclusion criteria were overt neurogenic bladder dysfunction, urodynamically confirmed bladder outlet obstruction, prior pelvic surgery or radiation, malignant diseases of the pelvic organs, anti-incontinence surgery, urinary tract infection (UTI), and any other serious diseases making the patient unsuitable for the study, as considered by the investigator.

Patients were treated with suburothelial injections of 100 U of onabotulinumtoxinA (Allergan, Irvine, CA, USA) in 10 mL saline, 0.5 mL per injection in 20 injections in the bladder body, sparing the trigone. All procedures were performed transurethrally by a single urologist (HCK) under light intravenous general anesthesia in the operation room. Anticoagulant use was discontinued 1 week before onabotulinumtoxinA treatment. Bladder volume was maintained at 100–150 mL and blood vessels were avoided during the injections. The onabotulinumtoxinA solution was injected into the urothelium at the posterior and lateral walls of the bladder by using a 23-gauge needle and rigid cystoscopic injection instrument (22 Fr, Richard Wolf, Knittlingen, Germany). After the onabotulinumtoxinA injection, a 14-Fr urethral Foley catheter was inserted and left for one day. The patients were discharged on the following day. Broad-spectrum prophylactic antibiotics were administered postoperatively for 7 days.

All patients were evaluated at baseline, 2 weeks, and 1, 3, and 6 months after treatment. Treatment results were assessed using the Global Response Assessment, which is categorized as −3, −2, −1, 0, 1, 2, and 3 indicating markedly worse, moderately worse, mildly worse, no change, mildly improved, moderately improved, and markedly improved bladder symptoms, respectively. The OAB Symptom Score, Urgency Severity Score, Patient Perception of Bladder Condition, and voiding diary parameters including daytime frequency, nocturia, urgency, and urgency urinary incontinence episodes per 3 days (3 consecutive days within 7 days before the visit) were also evaluated. Additionally, uroflowmetry and PVR volume (Laborie®, Mississauga, Canada) were measured at each visit. The variables measured included maximum flow rate (Qmax), PVR volume, voided volume, and VE.

Procedure-related AEs were recorded during the 6-month follow-up period after onabotulinumtoxinA treatment. The common AEs included acute urinary retention (AUR) (severe difficulty urinating with a PVR volume >350 mL and necessitating the use of an indwelling catheter or clean intermittent catheterization); PVR volume > 200 mL (without requiring an indwelling catheter); gross hematuria; general weakness; and UTI (symptomatic or asymptomatic with a white blood cell count > 10/high-power field on urinalysis) during the follow-up period. Patients who developed AUR or PVR volumes > 350 mL were advised to perform clean intermittent catheterization to evacuate their bladders as needed.

Continuous variables were expressed as means ± standard deviations, and categorical data were expressed as numbers and percentages. Statistical comparisons between the groups were conducted using Fisher’s exact test for categorical variables and analysis of variance test for continuous variables. The Wilcoxon matched-pair signed rank test was used to compare the parameters before and after treatment. All statistical assessments were two-sided and considered significant at a value of *p* < 0.05. The statistical analyses were performed using SPSS version 15.0 statistical software (SPSS Inc., Chicago, IL, USA, 2006).

## Figures and Tables

**Table 1:** The parameters of patients with DHIC and OAB at baseline and two weeks, four weeks, three months, and six months after 100 U onabotulinumtoxinA injection.

Parameters		Baseline	2 Weeks	4 Weeks	3 Months	6 Months
OABSS	DHIC	12.3 ± 2.0	11.1 ± 2.56 *	10.1 ± 2.77 *	10.1 ± 3.80 *	9.61 ± 3.24 *
	OAB	11.2 ± 2.9	9.48 ± 3.2 *	9.52 ± 2.96 *	9.19 ± 2.87 *	8.06 ± 3.3 *
USS	DHIC	4.0 ± 0	3.48 ± 0.87 *	3.40 ± 0.88 *	3.7 ± 0.92	3.28 ± 0.96 *
	OAB	3.62 ± 0.74	3.14 ± 1.1 *	3.29 ± 1.01 *	3.10 ± 1.0 *	3.06 ± 1.06 *
GRA	DHIC	0	1.19 ± 1.33 *	1.30 ± 1.38 *	1.10 ± 1.74 *	1.50 ± 1.47 *
	OAB	0	0.95 ± 1.28 *	1.43 ± 1.12 *	1.52 ± 0.81 *	1.72 ± 0.96 *
PPBC	DHIC	4.67 ± 1.77	3.90 ± 1.70	3.25 ± 1.65 *	3.15 ± 1.76 *	2.89 ± 1.75 *
	OAB	4.52 ± 1.66	3.10 ± 1.61 *	2.48 ± 1.47 *	2.81 ± 1.63 *	2.56 ± 1.29 *
UUI/3 days	DHIC	7.44 ± 9.51	6.67 ± 11.1	5.84 ± 9.22	12.3 ± 20.9	9.53 ± 19.6
	OAB	6.0 ± 13.6	3.65 ± 7.39	4.70 ± 9.09	3.62 ± 8.13 *	2.88 ± 2.03 *
Urgency/3 days	DHIC	27.7 ± 17.7	27.9 ± 23.4	29.8 ± 30.2	27.5 ± 28.2	29.1 ± 33.5
	OAB	27.3 ± 15.0	22.4 ± 14.5 *	19.9 ± 15.7 *	26.9 ± 19.8	15.1 ± 11.9 *
Frequency/3 days	DHIC	26.6 ± 14.2	30.0 ± 14.4	31.1 ± 24.6	28.6 ± 22.4	29.7 ± 28.9
	OAB	38.0 ± 12.9	37.6 ± 22.5	31.5 ± 9.27 *	36.5 ± 19.8	31.4 ± 12.3 *
Nocturia/3 days	DHIC	10.5 ± 5.41	9.39 ± 3.29	8.16 ± 3.01	8.50 ± 2.09	7.59 ± 3.12 *
	OAB	11.4 ± 5.40	9.80 ± 4.57	8.55 ± 3.80 *	10.5 ± 5.0	9.44 ± 3.01
Qmax (mL/s)	DHIC	12.6 ± 10.7	8.43 ± 4.23	8.62 ± 3.34	10.1 ± 5.32	11.2 ± 6.33
	OAB	12.9 ± 7.1	10.9 ± 7.9	14.2 ± 7.02	14.5 ± 8.54	12.9 ± 8.2
Voided volume (mL)	DHIC	146 ± 69	146 ± 97	171 ± 99	171 ± 125	199 ± 126
	OAB	187 ± 106	164 ± 136	212 ± 93	205 ± 96	185 ± 106
PVR volume (mL)	DHIC	109 ± 149	199 ± 118 *	179 ± 93 *	194 ± 150	123 ± 79
	OAB	11 ± 15	158 ± 184 *	147 ± 123 *	81 ± 75 *	75 ± 72 *
VE (%)	DHIC	57.3 ± 24.8	42.3 ± 24.8 *	48.9 ± 19.8 *	46.8 ± 26.8	61.8 ± 20.1
	OAB	94.6 ± 7.9	50.9 ± 29.4 *	59.1 ± 21.8 *	71.6 ± 20.1 *	71.2 ± 22.4 *

DHIC: detrusor hyperactivity and impaired contractility; OAB: overactive bladder; OABSS: overactive bladder symptom score; USS: urgency severity score; GRA: global response assessment; PPBC: patient perception bladder condition; UUI: urgency urinary incontinence; Qmax: maximal urinary flow rate; PVR: post-void residual; VE: voiding efficacy. * Significantly different from baseline.

**Table 2:** Comparison of the adverse events and therapeutic duration in patients with DHIC and OAB after 100 U onabotulinumtoxinA injection.

Adverse Events	DHIC (*n* = 21)	OAB (*n* = 21)	*p* Value
AUR	7 (33.3%)	3 (14.3%)	0.277
PVR volume > 200 mL	12 (57.1%)	7 (33.3%)	0.215
UTI	8 (38.1%)	4 (19.0%)	0.306
Gross hematuria	0 (0%)	1 (4.8%)	1.000
General weakness	1 (4.8%)	0 (0%)	1.000
Therapeutic duration (months)	4.9 ± 4.8	7.2 ± 3.3	0.03

DHIC: detrusor hyperactivity and impaired contractility; OAB: overactive bladder; AUR: acute urinary retention; PVR: post-void residual volume; UTI: urinary tract infection.

## Abbreviations

OAB	overactive bladder
UAB	underactive bladder
PVR	post-void residual urine
DHIC	detrusor hyperactivity and impaired contractility
VE	voiding efficiency
AE	adverse events
Qmax	maximal flow rate
Vol	voided volume
BOO	bladder outlet obstruction
DM	diabetes mellitus
DU	detrusor underactivity
